# Orthopædic Surgery

**Published:** 1904-05-14

**Authors:** 


### ORTHOPÆDIC SURGERY.

*(Concluded from page* 101.)

**Surgical Pathology of Genu Varum and Genu Valgum.**—In a paper read at the meeting of the American Orthopædic Association Dr. Walter Blanchard[8] said that a case of genu varum usually exhibited three contributing curves in the following order of development :—(1) Exaggeration of the normal outcurve of the lower end of the femur ; (2) an out-bend of the upper part of the tibia ; (3) a distributed outbend of the tibial shaft. The least amount of overcorrection was required at the culminating point to give a straight and lengthened leg. In genu valgum the primary inward bend was usually in the upper part of the tibia, the succeeding inward curve being distributed through the shaft of the tibia. In this case the least over-correction was necessary at the apex of the deformity in the tibia. Dr. Blanchard had tried the operation of epiphysiolysis, following Renier's directions, but considered osteotomy or rapid osteoclasis superior modes of treatment, epiphysiolysis presenting greater risks, and the period of recovery being longer.

**Treatment of Paralytic Talipes by Tendon Transplantation.**—At a meeting of the American Orthopædic Society a paper was read on this subject by Dr. Royal Whitman,[9] of New York, in which he expressed the opinion that the operation had not proved so effective as was anticipated. The primary object of treatment was to prevent deformity, this being intimately connected with the function of the part ; yet, even when this operation was performed in paralysis of the anterior group of muscles, with lateral distortion, to which it was specially applicable, there was usually a recurrence of the deformity, the most constant cause of failure of the treatment being the mechanical disadvantage at which the transplanted muscle must do its work. In deformity of the varus type the foot should be placed in abduction. He considered astragalectomy, with backward diplacement of the foot upon the leg, with the removal of the cartilaginous surfaces, the most useful operation in deformities of the calcaneovalgus or varus type. The lengthened tendo-Achillis might be shortened as a supplementary measure.

**Club Foot.**—Ogston[10] describes a new method of treatment for severe cases of this deformity in children under seven years of age. For cases not dealt with in the early stage he recommends Phelps's method of open incision ; in severe cases Lund's operation of excision of the astragalus, with tenotomy of the tendo-Achillis, but considers that his new plan involves less loss of bony substance. The tarsal bones in young children being composed of an outer thick layer of cartilage and an inner rigid kernel of bone, he proposes to open into the bones, remove the kernel in whole or in part by means of a sharp spoon, and so be able to mould the foot into shape, keeping it in position by plaster. Ossification will go on in the bones in the corrected position, leaving a strong and well-formed foot. Several tarsal bones may be dealt with by one incision. Photographs of cases treated in this way show satisfactory results, and the method seems to promise well.

**Phelps's Operation for Club Foot.**—This method of treatment consists in the open section of all resistant tissues on the inner side of the sole of the foot in every case of congenital club foot which is not completely cured by milder methods at the age of four months. Mr. Muirhead Little[11] has performed this operation 27 times since 1900, and gives an account of his experience. The operation is performed in the following manner :—After the usual antiseptic precautions have been taken, the patient is laid on the affected side, the foot resting on its outer border and dorsum. A V-shaped incision is made through skin and fat, with its base towards the outer border of the foot, about one-fourth of the width of the sole away from that border, with its posterior limb close in front of the internal malleolus, and its apex over the tuberosity of the scaphoid. This flap is reflected, and all the soft parts beneath it which resist forcible attempts at correction of the deformity divided, avoiding the external plantar vessels and nerve. If the foot cannot be straightened, the neck of the astragalus may be divided by chisel, but this is seldom desirable. The tendo-Achillis is divided subcutaneously, all bleeding arrested, the flap laid across the wound, and its end secured by one or two silkworm gut sutures. It will be found that in the corrected position of the foot there are two narrow fusiform open wounds, which will probably need skin-grafting later on. A sponge, gauze, and wool dressing are applied, and the foot and leg placed upon a wooden back splint, with the sole flat and the front part of the foot well abducted. The dressings are changed in a few days, skin grafts applied to the granulating wounds, and the foot and leg put up in gypsum in the best obtainable position. This proceeding differs from that described by Phelps in the following particulars :—(1) The tendo-Achillis is divided last, its resistance being useful in the manipulation of the sole during operation ; (2) the skin flap, which shortens the existence of an open wound, and prevents an unsightly scar ; (3) the foot is not put up into gypsum immediately after the operation. The immediate results have been in all cases good, and the later results satisfactory to the patients. Mr. Little considers that in selected cases Phelps's operation is of great value, but a skin flap should always be made ; that osteotomy as a part of the operation is rarely necessary ; that the operation is contra-indicated in young children, owing to the difficulty of predicting the ultimate result of growth and pressure, and the danger of severe flat foot following ; and that if for reasons of expediency cases must be operated on young, and flat foot result, the patient is better off than if he had relapsed or partly cured varus.

[8] Med. Rec., Aug. 15, 1903, p. 273 [9] Ibid., p. 274. [10] Brit. Med. Jour., June 21, 1902. [11] Ibid., Oct. 17, 1903, p. 977.

